# The Lathe

**Published:** 1848-01

**Authors:** A. M. Leslie


### THE LATHE, BY A. M. LESLIE, D. D. S.

One of the most useful instruments in the Dental Laboratory I believe to be a good Foot Lathe. Not simply something which will run an emery wheel and polishing brushes, but a complete Lathe, one on which small articles of wood and metal may be turned, to suit the various objects I purpose in this article to point out. Such a Lathe I would have small, but it must be *well made*, in order that the truth and permanency of its centres may be satisfactory. Perhaps I could not in a better way convey to the student about entering into practice my idea of what is well calculated for the work I propose than to describe the Lathe I make use of in my own Laboratory. It is not showy, but plain, substantial and neat.* It is mainly made of cast iron, the friction points, such as the journals, mandrils and centres, of course, are hardened steel. The shears are two feet, ten inches long; they meas-

* Such a one, with bench and a small case of drawers for the lathe tools, wheels and brushes, can be had for $35 or $40.

ure two and a quarter inches from centre to centre of edge; they are furnished with the wedge shaped edge, on which the heads and rests slide; which, as usual, are made stationary at any point on the shears, by means of a nut underneath. Inasmuch as but one rest generally accompanies a Lathe, and as an additional one is required to support a vessel with water to wet the emery wheels, I will here describe a very simple one, the materials of which, as well as the vessel it supports, happened to be in my box of sundries, or I might have had to describe a more costly, but certainly not a more useful article. The rest is formed of a carpenter's common gauge, one end of which has a screw nail with the head cut off and the sides of the uncut portion squared, driven into it; this screw is for holding a wooden nut, which plays under the shears and holds the rest at any desired point. That part of the gauge having the thumb screw in it rests upon the shears; the thumb screw is used to regulate the vertical motion of the support. For the vessel I made use of a section of the nozzle of a brass faucet—that portion where the curve is, at which portion there is a projecting piece, which formed the point of connection with the wood. This was done by setting it in the wood and drilling two pin holes through both, and with a common pin they are firmly united; on that end of the vessel from which the wheels revolves, fasten a sponge by means of a pliable wire, say copper, which allows of its adjustment to the different wheels and stones. I place water in the vessel in which the edge of the wheel revolves, the sponge effectually preventing the water from being thrown off. The bench on which the Lathe stands should be framed together firmly, be a little longer than the shears, and be two feet wide, which will give room for a small case of drawers behind the Lathe. The wood used for the bench should be two inches thick. A bench, such as described, when placed on a level floor will not shake, which is a point not to be overlooked. A mistake which I have frequently found made by Dentists, is the selection of a small driving wheel. As motion is what we want more than power, the wheel should be large, say thirty inches in diameter and heavy at the circumference. A one and a half inch crank is powerful enough for work suited to the foot, and the less lift the foot makes the easier it is for the operator. When it is desired to increase the power, some have resort to lengthening the strap or cord, so as to suit it to the larger parts of the pulley, but the method I adopt is what machinists term suspending the crank upon a gallows. This consists of two vertical pieces of wood two feet long and three inches wide, mortised into a horizontal piece three inches square, its length being the distance between the legs of the bench, measuring in a line with the Lathe; there are two pieces on each vertical piece, placed at right angles to and sunk half into it, the other half being allowed to play between the legs of the bench and on their inner surfaces, which serves to keep the gallows in its place. The centres which suspend the crank are screwed into the vertical pieces, the whole of which is elevated or depressed by the agency of a cabinet maker's hand-screw, which passes through but does not screw into the bench, the head of it merely resting on it; a thread is cut in the horizontal piece, in which the screw plays. The mandril on which the pulley is fixed has a screw thread cut on its free end; on this the metal chucks fit, of which there should be at least three, one carrying a centre, another a small screw in the centre of a plate, for turning small wooden articles, and the third should have a hole drilled through its centre, three-eighths of an inch in diameter, with another in its side intersecting the first. This chuck is designed for carrying drills, but I make good use of it for other purposes. There should be a small gouge and chisel for turning wood, a half oval and a square graver for turning metal, a small but very fine chisel for cutting burrs, to which add a centre punch and a catch and you are prepared to make and use a few articles very useful in finishing a whole or part of a sett of teeth—they will expedite the work very much, give it as high a finish with less labor, and strain the work less than any other method with which I am acquainted. In running the emery wheels, brushes and articles for finishing, I prefer to do so without using the right hand centre, which when used increases the friction very much. I am also thus enabled to place the hand rest at a right or any other angle to the axis of the mandrill. Without this I could not make that use of the Lathe I now do with much pleasure.

I had not long used my Lathe for fitting teeth before it occurred to me that its use might be increased by running a sett of burrs and drills to remove the superfluous solder from the rivet heads and backings of the teeth, or other portions of a piece of work. Although the idea was approved of by one or two of the students of 1846 to whom I then mentioned it, it is only recently I have verified its advantage; my attention in the meantime being successfully engaged in experimenting in other branches of Dental science, of which I will probably speak hereafter, should further experience prove their advantage to the profession. The burrs I make use of I formed from the handle of an excavator, which, of course, has first to be softened by heating and slow cooling before cutting, after which it must be heated and plunged into water or oil. This may make the postern of the drill which fits into the stock too hard, which may be remedied by holding that part in the flame of an alcohol lamp, or between the red hot beaks of a pair of tongs, until it assumes a blue color, when it should again be plunged into the water or oil. The burrs I make one inch long, one end of which fits into a stock similar in form, but of course larger than a watch maker's drill stock. The stock itself is fitted into the drill chuck. The form of the burr may be much varied, but I think the rose head, the rounded edge, the truncated cone, and the cylinder head will accomplish all that is desired; indeed, what I have termed the cylinder head will be found most useful; with the exception of the rose drill, the teeth are all cut on the circumference of the head, the ends being left smooth. The heads must of course be of various diameters and lengths. For the cylinder and cone, three-sixteenths of an inch I find suits for the largest size, as well as most of the work; between the burr and the stock the body should be turned down smaller, so as to leave the burr projecting as a head. For cutting the smallest burrs, a very fine file will answer a good purpose. I also find it very convenient to make use of the watch maker's drill stock and drills, which are small; they answer well for cutting off a tooth or the rivets of a broken one, when it is desired to replace it with another. The stock I insert into a wooden chuck, having previously taken off its pulley.

Useful as I have found this improvement to be since accomplished, it appeared far from complete without some method of stoning the work at the Lathe, so irksome had it always been to me to do it by hand; but the great difficulty in the way was the want of a stone with a true and durable point; a true point could easily be given to the Scotch stone commonly used by silversmith's but its softness entirely unfits it for stoning the more difficult points. Having previously made use by hand of that new mechanical agent which one manufacturer has styled Arkansite, but which is more extensively known as the Arkansas oil stone, for the purpose of stoning the backings of the teeth, I found it had the requisite durability of point, so indispensable in the Lathe; having ascertained this, my next step was to find a piece with a pencil point, which is the shape I make use of; but after searching over the largest stock and greatest variety I have yet seen in the west (which is kept by Messrs. Allen, dealers in watches, &c., Main st., 3d door below Fourth, Cin.,) the nearest approach I could make to it was a piece which measures an inch and a half in length, one-fourth inch broad and one-eigth in thickness, with a bevel on one end, which was no better than if it had been squared. All who have purchased a piece of this stone know that the cost of cutting is the reason given for the high price of some of the peculiar shaped pieces. I would willingly have given three or four dollars for such a piece as I wanted, but it was not to be had. How then to make the point I wanted was the trouble; I had not the lapidaries' wheel, with its steel, copper, lead or wooden disc, revolving horizontally, carrying emery and water with which to form it, and which, I doubt not, is the means made use of; albeit we hear something of the cost of diamond dust in connection with the preparation of the Arkansite. I had not these, I say, but every Dentist has that which does the work quite as well; that is, the emery wheel. This, of course, was the first thing I tried, and with which I not only made a point, but was enabled to make a perfectly true one, after fixing it in the chuck in which I use it. The knowledge of this simple fact enhances to me, and doubtless will to others, the value of this, to Dentists, incomparable stone; with it I am enabled to fashion small pieces of the stone so as to suit, when fixed in instruments prepared for the purpose, exceedingly well for the preparation of many stoppings for the burnisher which I could not reach with the best pieces I had formerly provided myself with. By means of the above named pointed piece of stone and a larger portion of Scotch stone, I am enabled to stone off every part of a piece of work at the Lathe in a much shorter time and a better manner than I can by hand. The Arkansite stone I have fixed into a chuck which is formed of a tough piece of wood (say sugar tree). It is two inches long, one-half of which is turned to fit tightly into the drill chuck; the other half is five-eighths of an inch in diameter. Into this end the stone is sunk and wedged there; the Scotch stone is cut down with a rasp so as to fit the drill chuck, one thickness of paper being put around it before insertion it will be found to hold well. In using it I prefer water, a small vessel of which should rest on the shears immediately under the stone, in which the piece of work may be dipped or washed, that it may be seen how the work proceeds. In using the Arkansite I make use of oil, as it prevents the gold from adhering to the stone; a piece of muslin should be kept for the purpose of wiping off the oil when it is desired to see how the work goes on; this cloth, when saturated with oil, together with the stonings found in the bottom of the vessel used with the other stone, should not be thrown away, but cast into a receptacle for old crucibles and the like, the returns from which will well pay for the one day's labor in a year devoted to this kind of mining, the method of doing which and of melting and refining gold and silver may probably form the topics of a future article. In using the burrs or stones for cleaning up a surface which is level, it is necessary to give the work a constant lateral or vertical motion, according as it is held.

In conclusion of this already much longer article than I designed writing, I will describe a simple method of attaching emery wheels to such a Lathe as I have described. I make for each wheel a wooden chuck, similar to that for the Arkansite stone, with the exception that the diameter of the free end varies; this end is cut off perfectly true in the Lathe; on it the wheel is placed, and held fast by a small screw nail, which passes through its centre into the wood, a portion of the bevelled part of the head being filed off so as to leave a flat shoulder to press against the wheel A wheel fixed thus, if made true will turn true, but as they are not always so, and it is essential they should be true, I would here say that the method I adopt is to select from among my porcelain tests one of the most thoroughly vitrified pieces I can find, which being held in a pair of pliers to the wheel without water while in rapid motion, the wheel will soon be found to become heated, when it may be turned off in ribbons if desired. A damaged tooth will answer when the other cannot be had; the pressure exerted when the wheel becomes heated should not be great, and a firm and steady rest for the hand is indispensable. The emery wheels should vary in size; my own run from one-half inch to five inches in diameter. The smaller ones I use most particularly in fitting the inner surfaces of gum teeth; the largest I make use of in fitting pivot teeth and the edges of the gum teeth, where the gum unites. For these purposes I use only the side of the wheel, which is very true and cuts a level base for the pivot teeth and edge for the gum, which is much more difficult with the circumference of any wheel. For carrying the brushes I use an iron chuck which fits into the drill chuck; it has a screw cut on its free end, on which the brushes all fit. There should be a variety of these, say from one and a half to four inches in diameter; they should have from one to four rows of bristles, the bristles of some inclined to an angle of forty-five to its plane; the remainder set in the plane, as is the case with most that are made. In brushing I make use of rotten stone and oil; this I follow with crocus and water, and finish off with the bnrnisher and dissolved soap.

The chucks should be made of thoroughly dried wood, but should they at any time be found too loose the simply wetting them in water before placing them in the chuck, will be found to make them very tight through the expansion of the wood.

DR. TAYLOR.

*Dear Sir*:—You are well aware of the advantage that it would be to the reader were this article accompanied with one or two illustrative drawings, but in the present stage of the Register's life I cannot think of asking the editors to be at that expense, and trust that those who may desire to try the value of it may, by close reading, be enabled to understand the several parts of things described. Respectfully yours,

Cin., Jan. 7th, 1848. A. M. LESLIE.

We hope when the Register becomes more firmly established that the society will authorize and be able to bear the expense of several neat engravings for each No. The branch appertaining to the Practice of Dentistry on which our friend has favored us with his communication, particularly requires this. We think, however, he has made it sufficiently plain to be perfectly understood without the aid of any drawing.

We can conceive of nothing more desirable in a Dentist's Laboratory than such a Lathe as is described by Dr. Leslie. The facility with which artificial work can be finished would well compensate for the cost and trouble of getting up such an instrument. Besides there are many small instruments which we almost daily need, either in our Laboratory or Office, which with a Lathe of this kind can be made by an ingenious Dentist much sooner and with less trouble than to superintend and have it done by a cutler.

The Laboratories of most of our Dentists are rather sorry looking affairs, scarcely possessing the first facility for getting up a substantial piece of artificial work. A Laboratory, to be complete, should be so supplied with a Furnace, Lathe, Rolling Mill, Benches, Hammers, &c., &c., that the whole process of putting up a full and complete denture can be done without going out to get the assistance of any instrument or individual. We therefore hail with pleasure any improvement in this department of our dental establishment.—Cin. Ed.

